# Update on the Epidemiology of Middle East Respiratory Syndrome Coronavirus (MERS-CoV) Infection, and Guidance for the Public, Clinicians, and Public Health Authorities — January 2015

**Published:** 2015-01-30

**Authors:** Brian Rha, Jessica Rudd, Daniel Feikin, John Watson, Aaron T. Curns, David L. Swerdlow, Mark A. Pallansch, Susan I. Gerber

**Affiliations:** 1 Division of Viral Diseases, National Center for Immunization and Respiratory Diseases, CDC; 2 Office of the Director, National Center for Immunization and Respiratory Diseases, CDC

CDC continues to work with the World Health Organization (WHO) and other partners to closely monitor Middle East respiratory syndrome coronavirus (MERS-CoV) infections globally and to better understand the risks to public health. The purpose of this report is to provide a brief update on MERS-CoV epidemiology and to notify health care providers, public health officials, and others to maintain awareness of the need to consider MERS-CoV infection in persons who have recently traveled from countries in or near the Arabian Peninsula.*

MERS-CoV was first identified and reported to WHO in September 2012 (1). As of January 23, 2015, WHO has confirmed 956 laboratory-confirmed† cases of MERS-CoV infection, which include at least 351 deaths. All reported cases have been directly or indirectly linked through travel or residence to nine countries: Saudi Arabia, the United Arab Emirates, Qatar, Jordan, Oman, Kuwait, Yemen, Lebanon, and Iran. In the United States, two patients tested positive for MERS-CoV in May 2014, each of whom had a history of fever and one or more respiratory symptoms after recent travel from Saudi Arabia (2). No further cases have been reported in the United States despite nationwide surveillance and the testing of 514 patients from 45 states to date.

The majority (504) of the 956 MERS cases were reported to have occurred during March–May 2014 (Figure). However, WHO continues to receive reports of MERS cases, mostly from Saudi Arabia.§ From August 1, 2014, through January 23, 2015, WHO confirmed 102 cases, 97 of which occurred in persons with residence in Saudi Arabia, including three travel-associated cases reported by Austria, Turkey, and Jordan; of the remaining cases, two cases were in persons from Qatar, and three cases were in persons from Oman.

CDC continues to recommend that U.S. travelers to countries in or near the Arabian Peninsula protect themselves from respiratory diseases, including MERS, by washing their hands often and avoiding contact with persons who are ill. If travelers to the region have onset of fever and symptoms of respiratory illness during their trip or within 14 days of returning to the United States, they should seek medical care. They should call ahead to inform their health care provider of their recent travel so that appropriate isolation measures can be taken in health care settings. Health care providers and health departments throughout the United States should continue to consider a diagnosis of MERS-CoV infection in persons who develop fever and respiratory symptoms within 14 days after traveling from countries in or near the Arabian Peninsula, and be prepared to detect and manage cases of MERS.

Recommendations might change and be updated as additional data become available. More detailed travel recommendations related to MERS, including general precautions posted by WHO for anyone visiting farms, markets, barns, or other places where animals are present, are available at http://wwwnc.cdc.gov/travel/notices/alert/coronavirus-arabian-peninsula. The website also lists more specific WHO recommendations for persons with diabetes, kidney failure, or chronic lung disease, and immunocompromised persons, that include avoiding contact with camels.¶ Guidance on the evaluation of patients for MERS-CoV infection, infection control, home care and isolation, and clinical specimen collection and testing is available on the CDC MERS website at http://www.cdc.gov/coronavirus/mers/index.html. Treatment is supportive; no specific treatment for MERS-CoV infection is available. WHO has posted guidance for clinical management of MERS patients at http://www.who.int/csr/disease/coronavirus_infections/InterimGuidance_ClinicalManagement_NovelCoronavirus_11Feb13u.pdf?ua=1.

## Figures and Tables

**FIGURE:**
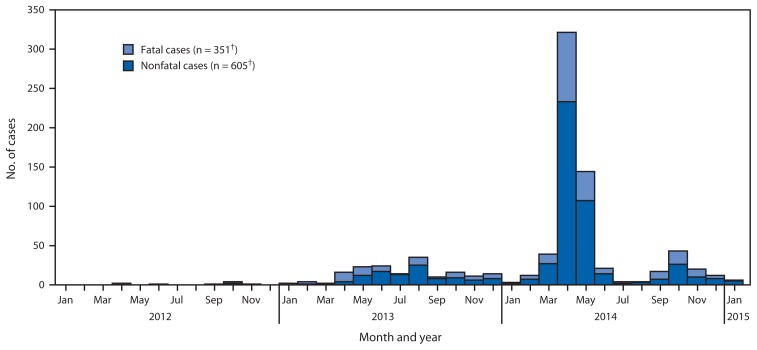
Number of cases of Middle East respiratory syndrome coronavirus infection reported by the World Health Organization,* by month of illness onset — worldwide, 2012–2015 *Data as of January 23, 2015, available at http://www.who.int/csr/don/archive/disease/coronavirus_infections/en. ^†^During June 3–October 16, 2014, a total of 130 additional cases and 84 deaths were reported with insufficient information to determine month of onset. These cases and deaths are not included in the figure but are included in the total cases and deaths counts.

## Footnotes

* Countries considered in the Arabian Peninsula and neighboring include: Bahrain; Iraq; Iran; Israel, the West Bank and Gaza; Jordan; Kuwait; Lebanon; Oman; Qatar; Saudi Arabia; Syria; the United Arab Emirates; and Yemen.

† Confirmatory laboratory testing requires a positive polymerase chain reaction test result on at least two specific genomic targets for MERS-CoV or a single positive target with sequencing on a second.

§ Additional information available at http://www.who.int/csr/don/archive/disease/coronavirus_infections/en.

¶ Additional information available at http://www.who.int/csr/disease/coronavirus_infections/MERS_CoV_RA_20140613.pdf?ua=1.
